# Quantitative PCR in Epidemiology for Early Detection of Visceral Leishmaniasis Cases in India

**DOI:** 10.1371/journal.pntd.0003366

**Published:** 2014-12-11

**Authors:** Medhavi Sudarshan, Toolika Singh, Abhishek Kumar Singh, Ankita Chourasia, Bhawana Singh, Mary E. Wilson, Jaya Chakravarty, Shyam Sundar

**Affiliations:** 1 Department of Medicine, Institute of Medical Sciences, Banaras Hindu University, Varanasi, India; 2 Departments of Internal Medicine and Microbiology, University of Iowa and the VA Medical Center, Iowa City, Iowa, United States of America; US Food and Drug Administration, United States of America

## Abstract

**Introduction:**

Studies employing serological, DTH or conventional PCR techniques suggest a vast proportion of Leishmania infected individuals living in regions endemic for Visceral Leishmaniasis (VL) remain asymptomatic. This study was designed to assess whether quantitative PCR (qPCR) can be used for detection of asymptomatic or early *Leishmania donovani* infection and as a predictor of progression to symptomatic disease.

**Methods:**

The study included 1469 healthy individuals living in endemic region (EHC) including both serology-positive and -negative subjects. TaqMan based qPCR assay was done on peripheral blood of each subject using kDNA specific primers and probes.

**Results:**

A large proportion of EHC 511/1469 (34.78%) showed qPCR positivity and 56 (3.81% of 1469 subjects) had more than 1 calculated parasite genome/ml of blood. However, the number of individuals with parasite load above 5 genomes/ml was only 20 (1.36% of 1469). There was poor agreement between serological testing and qPCR (k = 0.1303), and 42.89% and 31.83% EHC were qPCR positive in seropositive and seronegative groups, respectively. Ten subjects had developed to symptomatic VL after 12 month of their follow up examination, of which eight were initially positive according to qPCR and among these, five had high parasite load.

**Discussion:**

Thus, qPCR can help us to detect significant early parasitaemia, thereby assisting us in recognition of potential progressors to clinical disease. This test could facilitate early intervention, decreased morbidity and mortality, and possibly interruption of disease transmission.

## Author Summary

Anthroponotic VL caused by *Leishmania donovani* in the Indian subcontinent accounts for 70% of the world burden of VL. Among the estimated 100,000 cases of VL acquired annually in India, 90% occur in the state of Bihar. Leishmania infection can result in either symptomatic or asymptomatic infection. *L. donovani* infection can also manifest as post-kala azar dermal leishmaniasis, a chronic cutaneous form thought to provide the reservoir for anthroponotic transmission of VL in regions endemic for this parasite species. We hypothesized that, in areas endemic for *L. donovani*, asymptomatic infections might also play a crucial role in disease transmission. This study describes use of quantitative PCR (qPCR) to determine the infection status in individuals living in an endemic region of India. We hypothesized that parasite load estimation by qPCR of peripheral blood cells among healthy individuals living in the endemic region might reveal the true frequency of infections through direct evidence of parasitemia. We reasoned this test would detect both asymptomatic non-progressors as well as asymptomatic individuals who will progress to fully symptomatic VL. Serologic testing by ELISA or DAT showed poor agreement with molecular detection of parasite DNA by qPCR, suggesting the tests differentiate between infection and immune response. Amongst ten healthy individuals who progressed to VL, only six were serologically positive whereas eight were initially qPCR positive, among whom five had high parasite loads in their blood. Thus, deployment of qPCR technique to estimate the presence and level of parasitemia in healthy individuals from Leishmania endemic regions may contribute to early case detection, thereby reducing morbidity and mortality. Consistent with the goals of the VL control and elimination program, this early intervention approach could help interrupt disease transmission.

## Introduction

The *Leishmania* spp. parasites of humans are endemic in 98 countries, and more than 350 million people are at risk of infection [1]. Leishmaniasis is a neglected tropical disease, and the most severe form visceral leishmaniasis (VL, also known as kala-azar) is fatal if untreated. VL is primarily an anthroponotic infection caused by *Leishmania donovani* in India, transmitted by the sand fly vector *Phelobotomus argentipes* [2], [3].The state of Bihar in India accounts for 90% of cases in the country [4]. A majority of infected individuals do not develop clinical illness [5], [6], [7]. According to a serology-based epidemiological survey, the prevalence of asymptomatic *Leishmania donovani* infection in Bihar is 110 per 1,000 persons, and the rate of progression to symptomatic VL is 17.85 per 1,000 persons [8]. The kinetics of parasite amplification during the progression from infection to disease is as yet uncharacterized. We have recently shown that a highly quantitative qPCR test of blood can track the decrease in parasite load during successful treatment of infection [9]. The current study was based on the hypothesis that the number or the kinetics of circulating parasites in asymptomatically infected individuals, as measured by qPCR, might provide the most sensitive early indicator of infected subjects apt to progress to full blown disease.

Alternate techniques to detect parasites in persons with VL include direct histological examination and/or culture of bone marrow and splenic aspirates. However these methods are not feasible for screening methods or epidemiological research due to their invasive nature. Serological methods are simple, non-invasive means of detecting specific antibodies, but it is already shown that there is a lack of correlation between serology and nucleic acid methods for parasite detection [10], [11], [12]. This could reflect the inability of serology to distinguish past from ongoing infection, and therefore might result in overestimation of the number of infected asymptomatic individuals.

A large proportion of infected individuals are reportedly asymptomatic according to both serology and PCR surveys in India and nearby endemic countries [13]. Recent epidemiological reports from Brazil, Spain, and France have shown that detectable parasite DNA is present in the blood of asymptomatic infected individuals [14], [15], [16]. qPCR based epidemiological studies in the Mediterranean region have described a threshold and reference value for asymptomatic infection [17]. A similar study from our population in Bihar suggested the equivalent of 5 *L. donovani* parasite genomes detected/ml of blood is the threshold for clinical symptoms of VL to occur [18]. Data from our prior work in India suggest that serologic status is not a good predictor of conversion to symptomatic VL. Indeed, only 3.48% of seropositive individuals converted to active VL, whereas the conversion rate was 2.57% among seronegative individuals from the same endemic region [19]. We therefore investigated the potential for molecular quantification of parasite genome equivalents in blood as a more sensitive measure of asymptomatic infection likely to progress to disease.

Early case detection and treatment are the most important control measures for Leishmaniasis. Thus, the inability to identify individuals with asymptomatic infection, and among these to discern the individuals that are likely to progress to disease, presents a problem for clinical management. In response to this need, the current study constitutes a comparison of qPCR, serological testing with direct agglutination test (DAT), and the rK39 ELISA as predictors of progression from asymptomatic infection to fully symptomatic VL. We performed this study in a population of individuals living in the highly endemic Muzaffarpur region of the state of Bihar, India.

## Materials and Methods

### Study site and sample collection

The work was carried out in the Department of Medicine, Banaras Hindu University, Varanasi and at its field site Kala-Azar Medical Research Centre, Muzaffurpur, Bihar and villages of Muzaffarpur district. The study was approved by the Ethics Committee of the Institute of Medical Sciences, Banaras Hindu University, the University of Iowa and the National Institutes of Health. The IRB at Banaras Hindu University is registered with the US NIH. Written informed consent was obtained from each participating individual.

### Sample collection

The study was carried out in villages of Muzaffarpur district, which is endemic for VL. To identify individuals who had recently seroconverted, an epidemiological sero-survey was performed for two consecutive years (2009 to 2012). Villages from which large numbers of VL cases originated were identified from hospital records at Kala Azar Medical Research Centre. The research team enrolled all consenting adults age 18 and above in these villages. In the first survey, serology was done using DAT and rK39 ELISA from figure prick blood collected on filter paper. All individuals who were seronegative on the first survey were selected for testing for seroconversion by DAT and rK39 during the second serosurvey conducted 12 months later. To extract blood leukocyte DNA, two ml of blood were collected in the citrate-containing tubes from 401 recent seroconverters (seropositive) as well as 1068 randomly selected seronegative individuals within 15 days of serologic test. Buffy coat cells were isolated, and were transported from Muzaffarpur on ice to the central laboratory in Varanasi and stored at −20°C until use. 36 nonendemic healthy person's blood were also taken for qPCR assay.

### Serological test (DAT/ELISA) and molecular test (qPCR)

Sera were eluted from filter papers containing finger prick blood and used to perform serology by DAT and rK39 ELISA as described previously [20], [21]. Individuals who are either DAT or rK39 ELISA positive were considered seropositive.

DNA was extracted using the QIAamp DNA mini kit (Qiagen, Hilden Germany) as per the manufacturer's instructions Only those DNA samples that had an optical density (OD) 260/280 ratio of 1.8–2.0 and an OD 260/230 ratio >1.5 by spectrophotometer measurements (ND-2000 spectrophotometer; Thermo Scientific, Waltham, MA, USA) were taken for qPCR experiments. The TaqMan based qPCR assay was performed in a final volume of 10 µL containing 5 µl TaqMan master mixture (2×) [Applied Biosystems (ABI), Carlsbad, CA, USA], 4 µl of DNA template and 0.25 µl (5 µM) of forward and reverse primer and 0.375 µl of probe (Integrated DNA Technologies, Coralville, IA, USA) (Table 1.). Primer-probe sequences are listed in Table 1. Amplification was conducted in a 7500 Real-Time PCR system [Applied Biosystems (ABI), Carlsbad, CA, USA]. The standard curve method for absolute quantification of parasite numbers was used as described previously [9]. All assays included no-DNA template controls, as well DNA from a negative control unexposed healthy subject. Cutoff values to consider a test positive were Cyclic threshold (Ct) value of 39. According to the standard curve, 0.001 parasite genome equivalents in the well corresponded to a CT value of 39.

**Table 1:** Taqman primers and probe for detection of *Leishmania* DNA in endemic healthy controls.

	Primers	Probe
kDNA4 forward	GGGTGCAGAAATCCCGTTCA	ACCCCCAGTTTCCCGCCCCG
kDNA4 reverse	CCCGGCCCTATTTTACACCA	

Sequences correspond to the kDNA4 minicircle DNA primers and probe [21].

### Statistical analysis

Data analysis was done by non parametric Mann-Whitney test using SPSS 16 (IBM, Somers, NY, USA) and Prism (Graph pad software).

## Results

**Figure 1:**
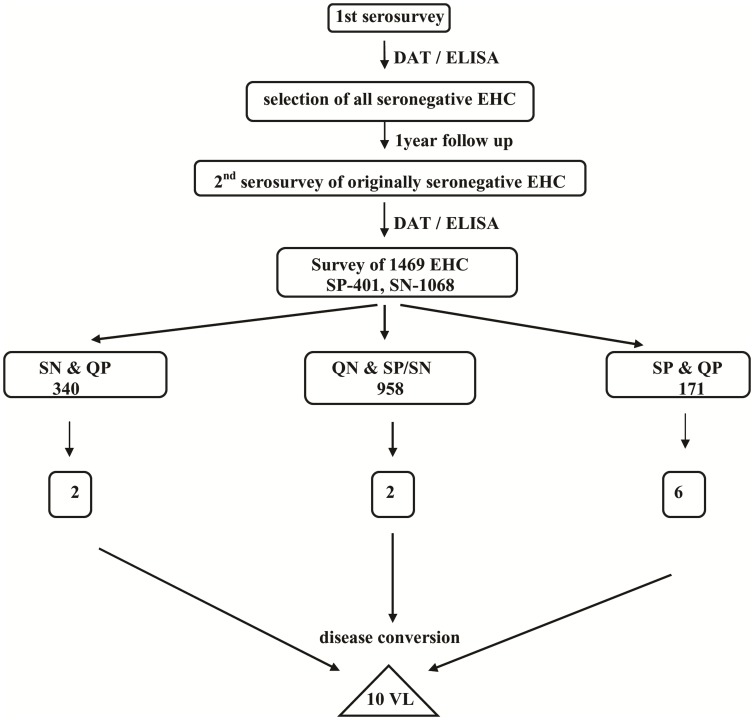
Flow chart of study of serology in endemic healthy control individuals (EHC) from baseline serosurvey (year 1) to identification of progressors. SP – seropositive; SN – seronegative; QP – qPCR positive (CT cutoff 39); QN – qPCR negative; VL – progressors to symptomatic visceral leishmaniasis.

A flow chart of the progression of results is shown in Fig. 1. Serological results were interpreted in light of our original description of the first serosurvey. Cutoff values for positive serology were chosen considering results from study of negative control unexposed Indian subjects, positive control subjects with acute or successfully treated VL, and recommendations from the serological test manufacturers [20]. Considering conversion of either the DAT or the rK39 ELISA as a seroloconversion, 401 subjects converted from seronegative to seropositive between the first and the second serosurvey, whereas the remaining 1068 individuals remained seronegative.

Quantitative PCR of DNA extracted from circulating blood cells was used to assess the proportions of individuals from the endemic region with evidence of asymptomatic parasitemia, and the correlation with serological conversion. The kDNA4 probe set and taqman assay was chosen from our previously published diagnostic criteria, because of the efficient amplification of *L. donovani* sequences and the lack of primer-dimers complicating quantification of low numbers of parasites [22]. Data were carefully controlled, and results of individual qPCR runs were only accepted when there was a lack of kDNA amplification in no-DNA and negative controls. A standard curve was run with each assay, using the same stock of promastigote DNA extracted from an Indian isolate, to ensure consistency between assays. Data were expressed as “genome equivalents” compared to this uniform DNA standard. Notably, more than four “genome” per ml were present in individuals with symptomatic VL according to our prior publication [9].

**Figure 2:**
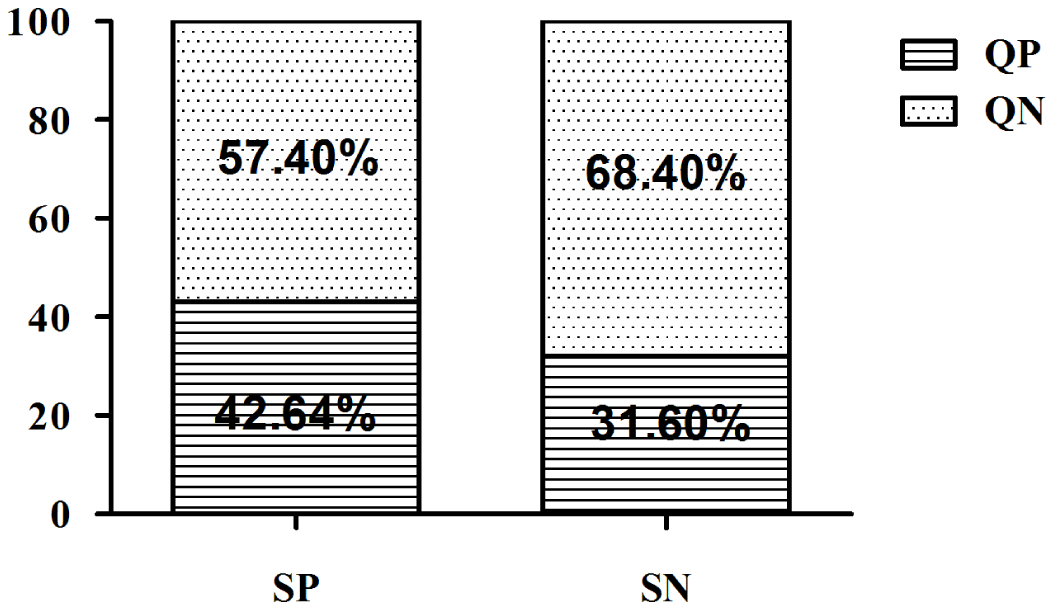
Bar graph to show Leishmania detected by qPCR in different group of EHC on the basis of their serological status (QP-qPCR positive, QN-qPCR negative, SP-sero positive, SN-sero negative).

Among a total of 1469 healthy individuals living in the endemic villages, 511 (34.78%) were positive by qPCR for amplification of any parasite DNA (CT less than 39). 171/401 (42.8%) were from seropositive group and the remaining 340/1068 (31.6%) were seronegative (Fig. 2, Table 2). The median value of parasite genomes/ml of blood was less than one and found to be 0.11 and 0.15 in seropositive and seronegative group respectively. Ten individuals who were initially belong to both seropositive and seronegative category progressed to symptomatic VL by the time of the follow up. Six (60%) were both qPCR and serology positive, two (20%) were qPCR positive but seronegative, whereas two (20%) were both qPCR and serologically negative (Table 3). Among these qPCR positive progressor five of the progessors had parasitemia levels equal to or more than the threshold value for ocuurence of symptomatic VL due to *L.donovani*.

**Table 2:** Parasitemia range in blood buffy coat samples from healthy controls from endemic or nonendemic regions.

	Range of parasite genome equivalents in blood cells from individuals with non-zero results of parasite qPCR test							
	positive	negative	>0–<1	1–5	5–10	11–100	101–1000	>1000
**Seropositive EHC** (n = 401)	171 (42.6%)	230	149 (**1** *)	16 (**1** *)	2 (1*)	1 (**1** *)	3 (**2** *)	0
**SeronegativeEHC** (n = 1068)	340 (31.8%)	728 (2*)	306 (**1** *)	24	5 (1*)	4	1	0
**Seronegative NEHC (n = 36)**	zero	all	-	-	-	-	-	-

(*converted into VL).

**Table 3:** Pogressors (disease conversion, VL cases) details: Parasite load and serostatus of EHC who were healthy at the first survey, but developed symptomatic VL at the time of the 12-month follow up.

S.No.	disease conversion time (after blood collection)	qPCR (genome equivalents/mlof blood)	Sero statuspos/neg
1	after one month	10.5	Pos
2	with in one month	6.6	Pos
3	after five months	97	Pos
4	after six months	0.38	Pos
5	with in one month	754.73	Pos
6	with in one month	146.32	Pos
7	after three months	5.09	Neg
8	after three months	0.613	Neg
9	after five months	0	Neg
10	after two months	0	Neg

(pos - positive on either serologic test; neg – negative on both serologic tests).

The non-randomness of qPCR results is illustrated by the fact that all noendemic healthy were negative for qPCR.

## Discussion

In this study *Leishmania* DNA was detected in large proportions of both seropositive and seronegative endemic healthy groups (Fig. 1). In contrast, to nonendemic healthy who were negative for the test. Similar findings were reported in a study of *L. infantum* infection, a cause of VL in the Mediterranean and in Latin America [17]. In one of our earlier study we showed that the parasite load in individuals with acute symptomatic VL due to *L. donovani* was at least 20, and at day 30 of treatment was >1.12 genome equivalents/ml [9]. In other study we found 5 parasite genome/ml of blood as the threshold value to differentiate asymptomatic from symptomatic [18]. Mary et al. cited a persistent level of more than 1 parasite/ml as a risk for relapse of *L. infantum* disease [17]. Our ability to quantify the parasite load in asymptomatic individuals led us to examine a potential threshold for progression to active infection in previously uninfected individuals.

A positive serologic test for *L. donovani* in individuals living in endemic regions who have no symptoms of VL could indicate prior exposure without substantial active infection, ongoing asymptomatic infection which will not lead to disease, or early infection that will progress. In this situation it would be extremely valuable to perform additional diagnostic testing that could be used as a marker of infection, and also be capable of differentiating those likely to progress from those at low risk for progression to disease. Given our results suggesting the magnitude of parasitemia is related to the risk of disease, a quantitative test such as the qPCR reported herein represents a candidate test for this distinction.

Both our study and the reported study of *L. infantum* parasitemia cite very low numbers of parasite genome equivalents in the blood as indicative of infection. It is important to appreciate the distinction between the calculated number of parasite genomes is not equivalent to the actual number of parasites in a ml of drawn blood. We previously reported that the number of kDNA copies varies between amastigotes and promastigotes, and that copy number is highly variable between strains of the same parasite species [22]. Given this variability as well as the fact that there is an anticipated loss of DNA in the extraction process itself, one can assume that the numbers of genomes quantified on a standard curve will be relatively quantitative compared to comparison samples treated in the same manner. However one cannot draw conclusions about the absolute numbers of parasites present in the subject based on these relative numbers. It is nonetheless important to use a standard DNA so as to obtain as equivalent quantitative measures between assays as possible.

A study of asymptomatic *L. donovani* infection in Nepal cited poor agreement between serological and molecular tests, i.e. DAT and routine PCR [5]. Our study similarly showed a lack of agreement between serology and qPCR (k = 0.1303). Herein 42.8% or 31.6% of healthy subjects from the endemic neighborhood who were seropositive or seronegative, respectively, contained *Leishmania* specific DNA in their blood (Fig. 2.). Potential reasons that seropositive individuals might become qPCR negative could include degradation and clearance of *Leishmania* DNA after infection, corresponding with development of protective immunity. A positive qPCR test in seronegative individuals could occur if the individual was bitten by a *Leishmania* infected sand fly, but either immunity has not yet developed or antibody levels are too low to be detectable by the methods employed. Analogous to infection with hepatitis B, it is possible that parasite DNA, detected by PCR of peripheral blood, could be the first marker of the infection prior to antibody seroconversion. Consistent with this hypothesis, during canine VL, kDNA-PCR is significantly more sensitive than the other parasitological and serological methods, allowing the identification of infected dogs even before the appearance of antibodies [23].

Quantification on the standard curve revealed that among qPCR positives, 56 subjects (10.95% of total qPCR positive) had more than one parasite genome/ml of blood, and among them 20 (3.91%) had five or more parasites (Table 2.). Although progression to disease occurred both in seropositive and seronegative groups, 8/10 (80%) of those converting to clinical VL were qPCR positive and 5/10 (50%) had relatively high parasite loads. This suggests that asymptomatic individuals who have high parasite load may be more likely to progress to disease than individuals whose parasite loads are low (Table 3.). Other reports of asymptomatic infection suggest that parasite DNA does not often persist for more than one year, but that rarely detectable asymptomatic infection may last for decades [24]. Further their follow up is necessary to know their conversion into symptomatic cases or they remain asymptomatic.

Our recent serological study from same population area shows there is an increased risk of progressing to disease among individuals with high titers of DAT or rk39 serology [21]. Although our study suggested that DAT/ELISA titers are less sensitive and specific than qPCR with high parasite load for detection of progressors, neither approach was perfect. It may be that a combination of qPCR to detect the presence and quantity of parasite nucleic acid, coupled with serology to identify individuals with very high titers, may be a practical and sensitive means of detecting infection, for use in early case detection. The qPCR measure serves as well as an effective tool to monitor clinical management. Early case detection and treatment are the most important control measures for leishmaniasis. In anthroponotic leishmaniasis in which humans are the only reservoir, early detection by qPCR should also be explored as a means of identifying individuals who might also pose a reservoir for disease transmission.

Limitations of qPCR include high initial investment, relatively higher cost per test compared to serology. Requirement of skilled personnel can be another limiting factor, however, if completely equipped and manned central laboratories are established at strategic locations to cater to one or several districts, a reliable diagnosis can be provided to population living in endemic regions for VL which will give more possibility of identification of symptomatic condition of VL disease in infected persons.

## Supporting Information

S1 Checklist **STROBE Checklist.**
(DOC)
